# Effect of C Addition on the Microstructure and Fracture Properties of In Situ Laminated Nb/Nb_5_Si_3_ Composites

**DOI:** 10.3390/ma16165637

**Published:** 2023-08-15

**Authors:** Delu Zeng, Lairong Xiao, Shaofu Xu, Huali Yu, Yu Zhang, Chenxu Yu, Xiaojun Zhao, Zhenyang Cai, Wei Li

**Affiliations:** 1 School of Materials Science and Engineering, Central South University, Changsha 410083, China; csuzdl@126.com (D.Z.); xiaolr@csu.edu.cn (L.X.); csuxsf@163.com (S.X.); 203112092@csu.edu.cn (H.Y.); zhangyu118@csu.edu.cn (Y.Z.); yu.chenxu@cxtc.com (C.Y.); zhaoxj@csu.edu.cn (X.Z.); csuczy@126.com (Z.C.); 2 Key Laboratory of Non-Ferrous Metal Materials Science and Engineering, Ministry of Education, Central South University, Changsha 410083, China; 3 Powder Metallurgy Research Institute, Central South University, Changsha 410083, China

**Keywords:** laminated Nb/Nb_5_Si_3_ composite, spark plasma sintering, orientation relationship, fracture toughness, compressive strength

## Abstract

//Nb_ss_ and α-Nb_5_Si_3_ phases were detected. Meanwhile, Nb_2_C was observed, and the crystal forms of Nb_5_Si_3_ changed in the C-doped composites. Furthermore, micron-sized and nano-sized Nb_2_C particles were found in the Nb_ss_ layer. The orientation relationship of Nb_2_C phase and the surrounding Nb_ss_ was [001]_Nbss_//[010]_Nb2C_, (200) _Nbss_//(101) _Nb2C_. Additionally, with the addition of C, the compressive strength of the composites, at 1400 °C, and the fracture toughness increased from 310 MPa and 11.9 MPa·m^1/2^ to 330 MPa and 14.2 MPa·m^1/2^, respectively; the addition of C mainly resulted in solid solution strengthening.

## 1. Introduction

The Nb_5_Si_3_ intermetallic compound has been considered as a potential material for high-performance structural application due to its high melting point (2520 °C), low density (7.1 g/cm^3^), and excellent strength retention at elevated temperatures [1,2,3]. However, due to the relatively low fracture toughness of 1–3 MPa·m^1/2^ at ambient temperatures [4], a ductile niobium-based solid solution (Nb_ss_) was brought into Nb_5_Si_3_ to achieve a balance of low fracture toughness and high temperature strength [5,6,7,8,9,10,11,12]. As for the as-cast Nb-Si alloy, with the increase in Si content, the volume fraction of ductile Nb_ss_ phase decreased, thereby significantly lowering the fracture toughness [13,14]. It was reported that the fracture toughness of Nb-10Si and Nb-16Si alloys were 12 MPa·m^1/2^ and 4.5 MPa·m^1/2^, respectively [15]. In order to improve the fracture toughness of Nb-Si alloys, a number of reinforcement elements such as Ti, Mo, and B were added as well [16,17]. Wang et al. [18] reported that the fracture toughness of Nb-16Si alloy improved after increasing the Hf content. Furthermore, the addition of B could also enhance the fracture toughness of a Nb-10W-10Si alloy [19].

Preparing unidirectionally solidified alloys and laminated composites has also been shown to be an efficient method of enhancing fracture toughness. Ye et al. [20] found that excellent fracture toughness of 14.5 MPa·m^1/2^ and 18.7 MPa·m^1/2^ could be exhibited by unidirectionally solidified Nb-Si and Nb-Si-Ti alloys, respectively. A Nb_5_Si_3_/Nb/Nb_5_Si_3_ laminate with a relatively high fracture toughness of 7.1–11.5 MPa·m^1/2^ was fabricated by hot pressing the Nb_5_Si_3_ compacts and Nb foil at 1200 °C for 5 h [21]. However, since the thickness of the Nb_5_Si_3_ compacts and Nb foil were 4 mm and 0.25 mm, respectively, the fracture toughness apparently changed into the distance changes of notch from the Nb/Nb_5_Si_3_ interface. Thus, the thickness of Nb_ss_ and Nb_5_Si_3_ layers should be decreased, and the in situ laminated Nb/Nb_5_Si_3_ composite with micron-sized multi-layer structures was fabricated from the previous work [22].

Furthermore, it was reported that the Nb-16Si-10Mo-15W alloy could be strengthened by addition a solution to B in the Nb_5_Si_3_ phases [23,24,25]. Similarly, it was confirmed that a C atom could also dissolve in the Nb_3_Al phase [26,27,28]. Due to fact that the C atom has a smaller radius than the B atom, the strengthening effect of adding a solid solution to C in the Nb_5_Si_3_ would be probably better than that of B. Reports regarding the addition of C to a Nb/Nb_5_Si_3_ alloy are scarce in the open literature. It is unclear whether and to what extent the addition of C can improve the fracture toughness of a Nb/Nb_5_Si_3_ alloy. Therefore, the aim of this work was to prepare in situ laminated Nb/Nb_5_Si_3_ composites supplemented with C via spark plasma sintering, evaluate the effect of C on the microstructure and mechanical properties of the composites, and identify the strengthening and toughening mechanisms.

## 2. Experimental Procedure

**Figure 1:**
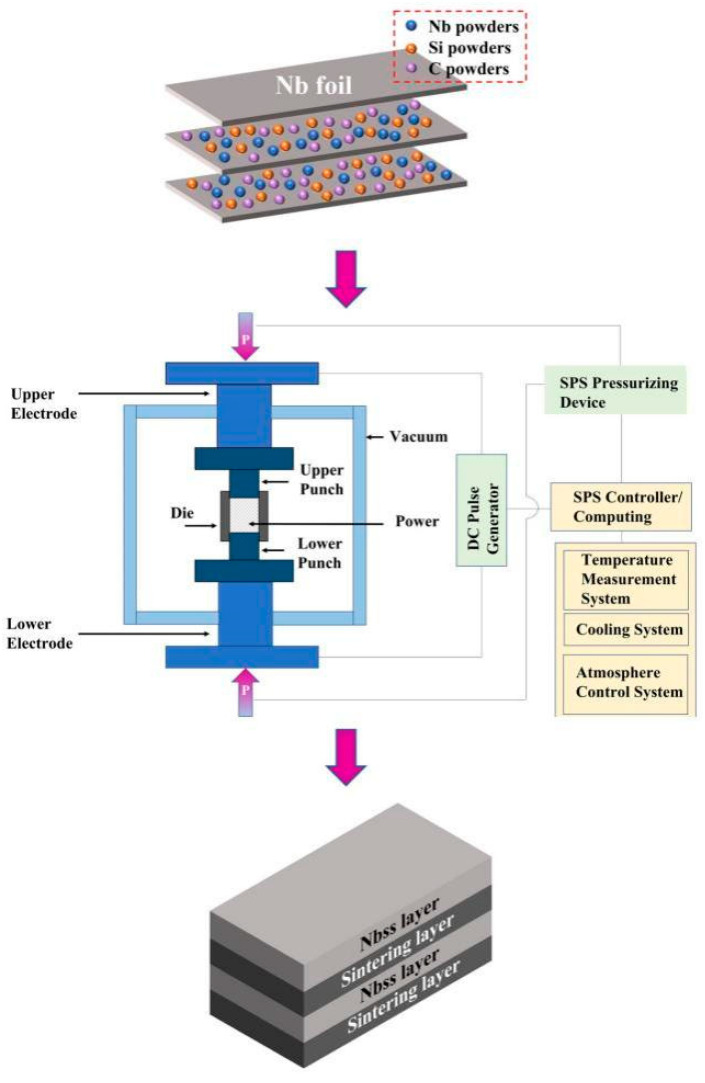
Diagram of the preparation process for the niobium-based composites.

Nb foils (99.99%, 25 μm in thickness), Nb powders (99.99%, 1–3 μm), Si powders (99.99%, 1–3 μm), and C powders (99.99%, 1–3 μm) were the raw materials used. For the experimental work, a three-step procedure was adopted. Firstly, mixtures of molar ratios of Nb-50Si and Nb-40Si-10C were selected to prepare the Nb/Si/(C) slurry via vacuum ball milling for 24 h using ethanol as a milling medium. For convenience of expression, the corresponding prepared materials are called Nb-50Si and Nb-40Si-10C, respectively. Secondly, the Nb foils were covered with mixed Nb/Si/(C) slurry via the dip-coating method and then stacked together and dried at 120 °C for 24 h in a vacuum. Thirdly, the stacked Nb foils were put in a graphite die and sintered at 1750 °C under a pressure of 30 MPa for 30 min in vacuum using a heating rate of 100 °C/min. Finally, the sintered Nb/Nb_5_Si_3_ composites were cooled at about a rate of 100 °C/min above 500 °C and then furnace-cooled down to room temperature. A diagram of the preparation process for the niobium-based composites is shown in Figure 1. The sintered material had a porosity of 0.3381% and a density of 8.1621 g/cm^3^.

The phase and crystallinity were analyzed via X-ray diffraction (XRD) using CuK_α_ radiation at 40 kV and 250 mA. The lattice parameters were calculated by using JADE 5 software. The microstructures of the samples were characterized using scanning electron microscopy (SEM), wavelength-dispersive spectroscopy (WDS), and transmission electron microscopy (TEM). The SEM samples were cut via electrical discharge machining (EDM) and polished to a surface finish using 1 μm diamond paste, and the TEM foils were prepared via ion milling. The volume fractions of phase in the composite were calculated via quantitative image analysis using EPMA micrographs; five EPMA images were used for each composite. Fracture toughness was determined via three-point bending (TPB) tests at room temperature. In the TPB tests, a specimen with a dimension of 2.5 mm × 5 mm × 20 mm and a notch introduced perpendicular to the layer direction was cut via electro-discharge machining (EDM), and the cross-head speed was set at a rate of 0.1 mm/min. Compression tests were conducted at 1400 °C at a strain rate of 10^−3^ s^−1^ in a vacuum. The dimension of the compression test specimen was φ4 mm × 6 mm, and the loading direction was parallel to the layer direction. Five specimens were tested for each condition, and the average values were recorded. A computer microhardness tester (200HBVS-30) was used to measure the Vickers hardness of the alloy under a load of 15 N, and the test time was 15 s. The same alloy sample was tested 5 times at random locations; a group of 3 samples were tested, and the average value of each measurement was taken as the Vickers hardness of the alloy.

## 3. Results and Discussion

**Figure 2:**
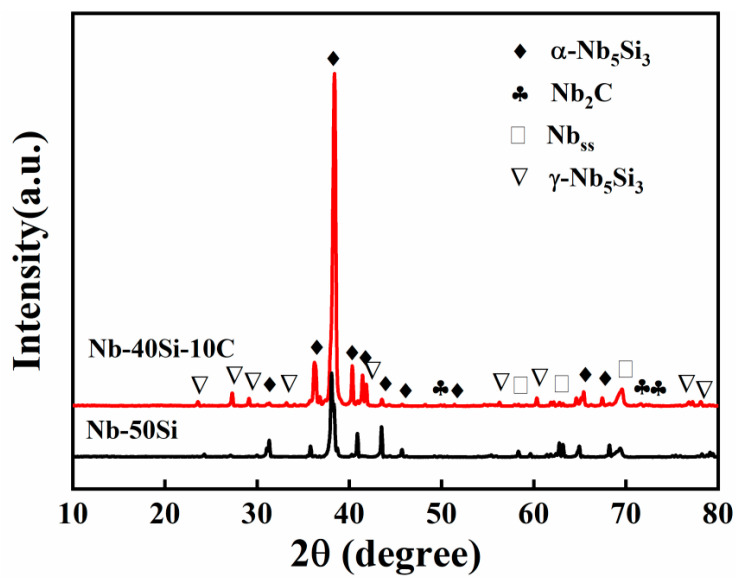
XRD patterns of alloys after sintering.

Figure 2 shows XRD patterns of the Nb-50Si and Nb-40Si-10C composites. It was found that the obtained Nb-50Si composite exhibited an XRD pattern typical of Nb_ss_ and α-Nb_5_Si_3_. However, following the addition of C, Nb_2_C and γ-Nb_5_Si_3_ were present in the Nb-40Si-10C composite. This indicated that the addition of C promoted the formation of the metastable γ-Nb_5_Si_3_ phase and high temperature β-Nb_5_Si_3_ phase. Additionally, it should be pointed out that SiC was not identified in the patterns. The PDF card numbers of the phases involved in the Figure 1 are shown in Table 1.

It was assumed that little SiC remained in the material. The following reaction was induced:

11Nb + 3SiC = Nb_5_Si_3_ + 3Nb_2_C (1)

The thermodynamic results of reaction (1) are listed in the following reaction (reaction (2)) according to the thermodynamic data shown in Table 2.

(2) $$∆H_{298}^{\Theta }=-817,134\;J\;\;\;\;\;\;∆S_{298}^{\Theta }\;=\;393.255\;J\cdot K^{-1}$$

The standard Gibbs free energy of reaction (1) can be expressed as (3), according to the calculation of the second approximation equation of thermodynamics:

(3) $$∆G_{T}^{\Theta }=∆H_{298}^{\Theta }-∆S_{298}^{\Theta }+∆C_{P}T\;(ln\frac{298}{T}+1-\frac{298}{T})$$

**Figure 3:**
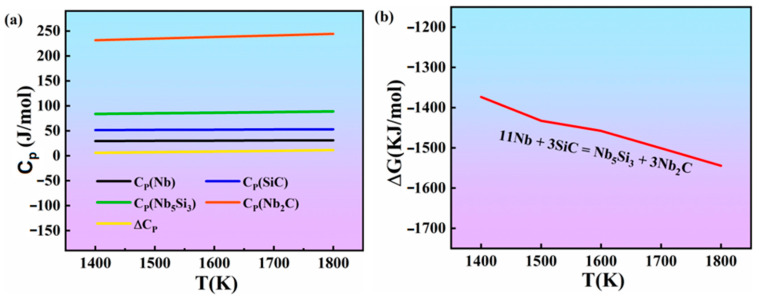
Thermodynamic data of the materials in reaction (1). (**a**) Molar heat capacity at constant pressure; (**b**) standard Gibbs free energy of the materials.

The molar heat capacities of the various substances in reaction (1) at 1400–1800 K and the standard Gibbs free energy at different temperatures is shown in Figure 3. As shown in Figure 3, with increasing temperature, the standard Gibbs free energy of the reaction (1) decreased, and all the values were negative. Therefore, according to the above results, reaction (1) occurred during the sintering process. Therefore, all of the XRD patterns show the absence of SiC in the Nb/Nb_5_Si_3_ composites.

**Figure 4:**
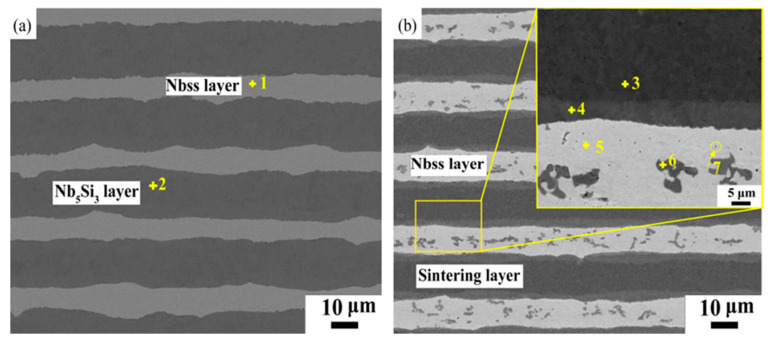
Microstructure of alloys: (**a**) Nb-50Si alloy; (**b**) Nb-40Si-10C alloy. Points 1–7 refer to the WDS punctual analysis summarized in Table 4.

Figure 4 shows the microstructures of the composites. Through WDS, alternately distributed Nb_ss_ layers (point 1 in Table 3) and niobium compound layers can be observed. In the Nb-50Si composite, the average thicknesses of the Nb_ss_ layers were decreased from 25 μm to 12.7 μm with increasing sintering time, confirm Si element diffusion from the Nb_5_Si_3_ layers (point 2 in Table 3) to the Nb_ss_ layers during sintering. Interestingly, with the addition of C, we observed that the microstructures of the composites significantly changed. According to the WDS results shown in Table 3, the Nb_2_C particle (3–5 μm) was present in the Nb_ss_ layers and exhibited a morphology different from the carbide in as-cast Nb-20Ti-12.5C-Mo-Hf alloys [29]. In addition, a lot of fine carbide (nano-sized) was also observed in the Nb_ss_ layer. The formation mechanism of the carbide will be discussed later. Secondly, both C-rich (point 4) and C-poor (point 3) Nb_5_Si_3_ were observed in the compound layers, which can be attributed to the different diffusion rates of the Si and C atoms in Nb. Due to the lighter atomic mass and smaller atomic radius of C compared to Si, the diffusion rate of C should be higher than Si, leading to the longer diffusion distance of the C element. As a result, the C-rich Nb_5_Si_3_ is closer to the Nb_ss_ layers. A relatively high oxygen content of 1.3 wt% was detected in the Nb_ss_ layer of the Nb-50Si composite. This was due to the fact that the dipping and stacking of the Nb foils was preformed in the air. Due to the fine Nb and Si powders, it is very difficult to avoid the physisorption of oxygen during the synthesis of the composites. However, interestingly, we observed that the O content (point 5) in the Nb_ss_ layer decreased to 0.3 wt.% in the Nb-40Si-10C composite.

The decrease in oxygen content can be explained via thermodynamic calculation. It is assumed that NbO exists in the material, and the following reactions can be assumed:

NbO + C = Nb + CO(4)

According to the relevant thermodynamic constants in Table 4, the thermodynamic calculation of reaction (4) is as follows:

(5) $$∆H_{298}^{\Theta }=298,236\;J\;\;\;\;\;S_{298}^{\Theta }\;=177.988\;J\cdot K^{-1}$$

**Figure 5:**
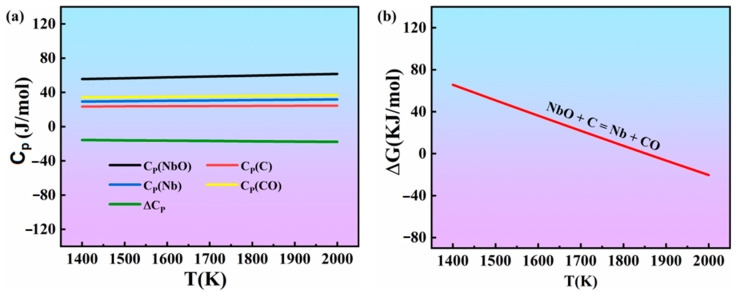
Thermodynamic data of the materials in reaction (4). (**a**) Molar heat capacity at constant pressure; (**b**) standard Gibbs free energy of the material.

Figure 5a shows the molar constant pressure heat capacities of the various substances in reaction (4) in the range of 1400–2000 K, which were calculated according to the second approximate equation of thermodynamics and allows one to obtain the standard Gibbs free energy of reaction (4) at different temperatures.

As can be seen from Figure 5b, with increasing temperature, the standard Gibbs free energy of the reaction decreases. When the temperature reaches 1900 K (~1627 °C), the standard Gibbs free energy is negative. As the sintering temperature of the alloy is higher than 1750 °C, reaction (4) can proceed smoothly with the sintering process, according to the above thermodynamic calculation results.

**Figure 6:**
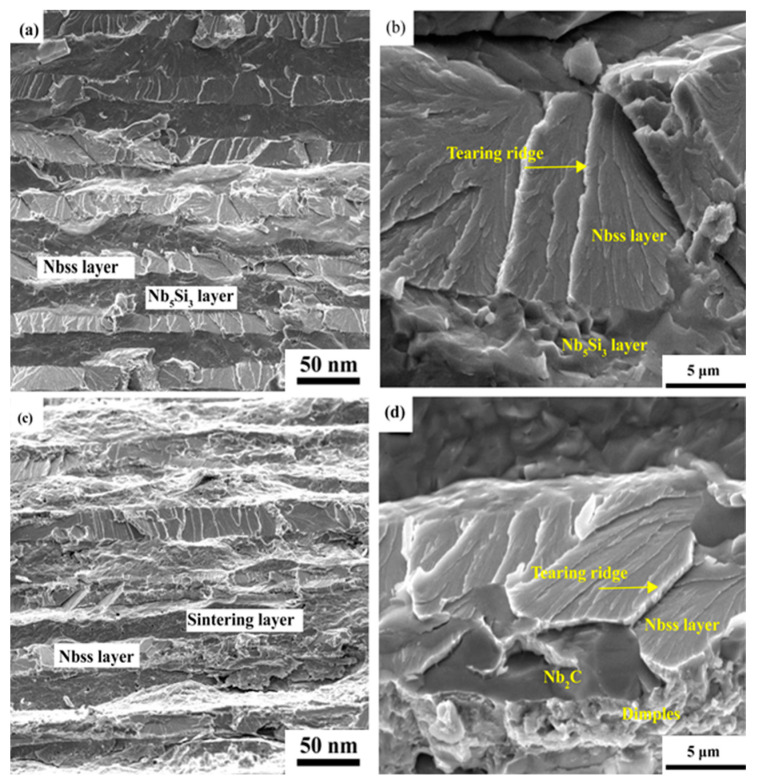
SEM images taken from the fracture surface of the alloys after the three-point bending tests: (**a**,**b**) Nb-50Si alloy; (**c**,**d**) Nb-40Si-10C alloy.

Figure 6 shows typical SEM micrographs from the fracture surfaces of sintered composites after the TPB tests. It can be seen from Figure 6a that the fracture surface of the Nb-50Si composite basically exhibited an intergranular fracture mode in conjunction with some cleavage fracture features in the Nb_5_Si_3_ layer. Meanwhile, transgranular cracking and some ridge-like features were observed in the Nb_ss_ layer. However, more transgranular cracking and some ridge-like features were observed due to the presence of the brittle Nb_2_C phase. This proved that the addition of C changed the fracture mechanisms of the composites. Additionally, regarding the Nb-40Si-10C composite (Figure 6b), partial dimples were observed in the Nb_ss_ layer.

In order to investigate the formation mechanisms of the Nb_2_C phase in the Nb_ss_ layer, the order of the reactions in the sintering layer was evaluated via thermodynamic calculation. As can be seen from Figure 4, three elements, namely Nb, Si, and C, were observed in the sintering layers. Furthermore, the C atoms tended to spread throughout the Nb_ss_ layers. Hence, what needs to be confirmed is whether the following two reactions occurred during the sintering process:

15Nb_2_C + 18Si = 6Nb_5_Si_3_ + 15C (6)

Nb_2_C + 4Si = 2NbSi_2_ + C (7)

According to the thermodynamic constants shown in Table 5, thermodynamic reactions (6) and (7) can be calculated as follows:

(8) $${∆H_{298}^{\Theta }}_{\left(6\right)}=298,236\;J\;\;\;\;\;∆{S_{298}^{\Theta }}_{\left(6\right)}\;=\;293.091\;J\cdot K^{-1}$$

(9) $${∆H_{298}^{\Theta }}_{\left(7\right)}\;=-81,170\;J\;\;\;\;\;{∆S_{298}^{\Theta }}_{\left(7\right)}=6.151\;J\cdot K^{-1}$$

**Figure 7:**
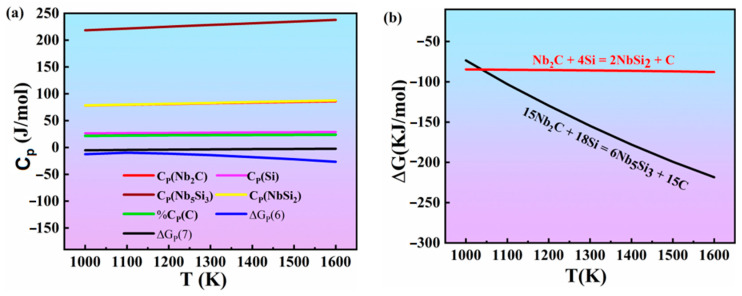
Thermodynamic data of the materials in reactions (6) and (7): (**a**) Molar heat capacity at constant pressure; (**b**) standard Gibbs free energy of materials.

The molar heat capacity $C_{P}$ of the various substances at 1000–1600 K are shown in Figure 7. Therefore, the standard Gibbs free energy $∆G_{T}^{\Theta }$ in reactions (6) and (7) at different temperatures could be obtained by using the second approximation equation of thermodynamics. It can be seen from Table 5 that, when the temperature was above 1000 K, the standard Gibbs free energy in reactions (6) and (7) decreased with increasing temperature. Furthermore, all of the values of the standard Gibbs free energy shown in Figure 7 are negative. This proves that reactions (6) and (7) could occur during the sintering process. In other words, even though Nb_2_C remained in the compound layers, it can react with Si and enter into C. It was indicated that a part of the carbon in the solid solution of Nb_5_Si_3_ and the others diffuses into the Nb_ss_ layers and then reacts with Nb, forming Nb_2_C.

**Figure 8:**
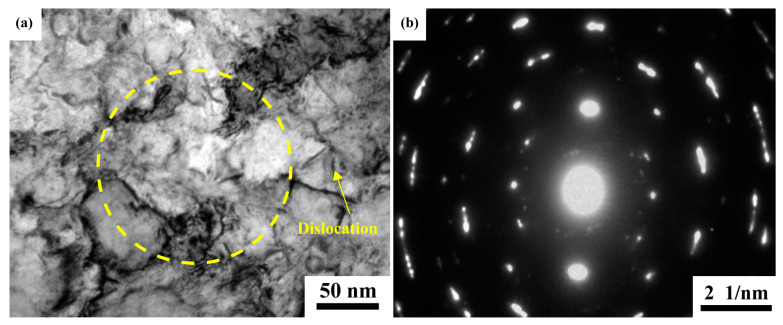
TEM images of Nb foil: (**a**) bright field image; (**b**) SADP of the region circled in Figure 8a.

Figure 8 shows TEM micrographs typical of Nb/Nb_5_Si_3_ composites. A large number of dislocations and other defects in the Nb foils are noted in Figure 8a. Figure 8b presents the region’s selection in Figure 8a, which is about 500 nm in diameter. The polycrystalline rings can be observed in Figure 8b, which indicates that there are multiple grains in this region. Therefore, it can be suggested that the grain size of raw Nb foil is below 500 nm and that there are many grain boundaries in the raw Nb foil, providing a channel for the diffusion of C atoms. During the sintering process, the C atoms rapidly spread into the Nb_ss_ and react with Nb in situ to form micron Nb_2_C particles in the Nb_ss_ layer.

**Figure 9:**
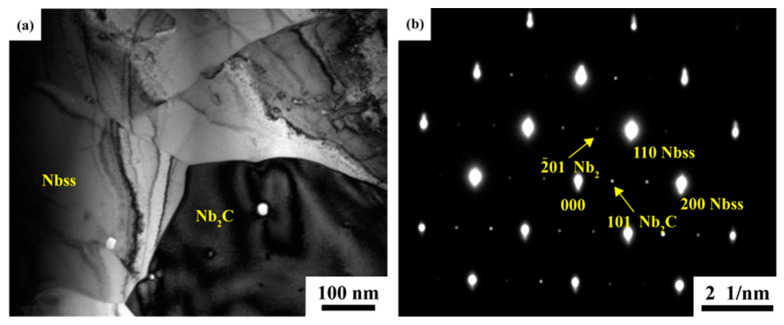
TEM images of Nb_2_C in Nb-40Si-10C alloy: (**a**) the Nb_2_C particle in Nb_ss_; (**b**) composite [010] SADP from Nb_2_C and [001] SADP from Nb_ss_.

Figure 9 shows TEM images of the micron Nb_2_C particles in the Nb-40Si-10C alloy and diffraction patterns of Nb_ss_/Nb_2_C. Nb_ss_ and Nb_2_C were observed in the Nb/Nb_5_Si_3_ composites, and the zone axes along [001] and [010] are presented, respectively. Furthermore, the phase relationship is [001]_Nbss_//[010]_Nb2C_, (200) _Nbss_//(101) _Nb2C_. The appearance of nano-sized Nb_2_C is mainly due to a change in the solid solubility of C in Nb_ss_. According to the Nb-C binary phase diagram [30], it is clear that when the temperature is above 1500 °C, the solid solubility of C in Nb_ss_ decreases considerably with decreasing temperature. Therefore, when the prepared Nb/Nb_5_Si_3_ composites are subjected to cooling at a sintering temperature of 1750 °C, a lot of nano-sized Nb_2_C can be precipitated from Nb_ss_.

**Figure 10:**
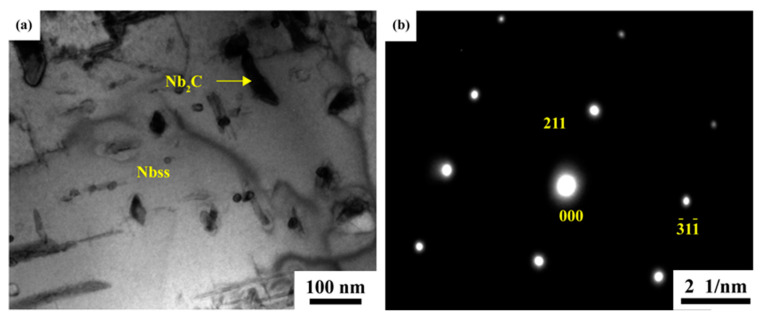
TEM images of nano-sized Nb_2_C in the Nb-40Si-10C alloy: (**a**) the nano-sized Nb_2_C precipitates in Nb_ss_; (**b**) [21$\bar{5}$] SADP from carbide in the precipitated Nb_2_C.

Figure 10 shows TEM images and diffraction patterns typical of nanometer Nb_2_C in the Nb-40Si-10C alloy. Club-shaped nano-sized Nb_2_C particles can be observed in Figure 10a, the length and width values of which are 100–300 nm and 70–130 nm, respectively. Nb_2_C was also observed in the Nb-40Si-10C alloy, and the zone axes along [21$\bar{5}$] are presented in Figure 10b.

The lattice constants of each phase can be obtained by analyzing and calculating the XRD patterns of the different components of the alloy using the Jade software (MDI Jade 6.0). Table 6 shows the lattice parameters of Nb_ss_ and α-Nb_5_Si_3_ in the composites. Regarding the Nb-50Si and Nb-40Si-10C composites, the lattice parameters of Nb_ss_ and α-Nb_5_Si_3_ decreased with the addition of C. The assumption that C atoms mainly occupy the substitutional sites in Nb_ss_ and α-Nb_5_Si_3_ can be confirmed by the fact that the atomic radius of C is smaller than that of Nb and/or Si, thereby forming a replacement solid solution.

The average compressive 0.2% flow stress at 1400 °C and fracture toughness at ambient temperature are shown in Table 7. It can be seen that the mechanical properties of the Nb/Nb_5_Si_3_ composites were significantly enhanced following the addition of C. This could be attributed to the following three reasons: Firstly, the C in Nb_ss_ and Nb_5_Si_3_ played a key role in solution strengthening and improving high-temperature strength. The dissolution of carbon atoms in both the Nb_ss_ and Nb_5_Si_3_ lattices was predominantly located at substitutional sites and decreased the lattice parameters, increasing the deformation resistance. As a result, the compressive strength is influenced by the content of the strengthened phase, i.e., Nb_2_C. As mentioned in Table 7, with the addition of C, the volume fraction of the plastic phase decreased, while that of the strengthened phase increased. Lastly, the precipitated fine carbide played a role in enhancing the compressive strength. Allameh et al. [31] reported that, with the addition of TiC particles, some dislocations in the TiC particles were observed, and it was also reported that their interactions played a significant role in strengthening the 44Nb-35Ti-6Al-5Cr-8V-1W-0.5Mo-0.5Hf (at.%) alloy. Therefore, it can be inferred that nanoscale Nb_2_C in the Nb/Nb_5_Si_3_ composites will produce similar strengthening effects.

It also can be seen from Table 7 that the fracture toughness of the composites improved with the addition of C. This can be attributed to the following reasons. First, as mentioned in Figure 4, the O content in the Nb_ss_ layer can be reduced or eliminated with the addition of C. This observation also corresponded well to the fracture morphology results shown in Figure 6. It is known that a large amount of energy could be absorbed from the plastic deformation of the Nb_ss_. When the plasticity of Nb_ss_ increased, more energy could be consumed, resulting in an increase in the fracture toughness of the composites. The ductility of Nb_ss_ can exhibit a strong resistance to crack initiation during the plastic deformation of 44Nb-35Ti-6Al-5Cr-8V-1W-0.5Mo-0.3Hf (at.%), as reported by Sikka and Loria [32].

Second, the fracture toughness can be affected by some physical properties. According to the Ashby model [33], the toughness increment Δ*K^C^* can be expressed as Equation (10):

Δ*K^C^* = (*C*·*V_f_* ·*E*·*σ*_0_·*a*_0_)^1/2^ (10)

where *E*, *V_f_*, *σ*_0_, and *a*_0_ are the Young’s modulus (GPa), volume fraction, yield strength at ambient temperature (MPa), and radius of the Nb_ss_ phase (m), respectively, and *C* is the material constant representing the degree of constraint imposed upon a ductile particle from the brittle matrix. In the current work, since the Nb_ss_ phase became deformed without interface decohesion (Figure 3), the parameter *C* is taken to be 1.6 [34]. The volume fraction and average radius of the Nb_ss_ can be obtained from Figure 3. The Young’s modulus and Vickers hardness were measured, and the yield strength *σ*_0_ (MPa) of Nb_ss_ phase can be estimated from the Vickers hardness of the Nb_ss_ phase using the following equation [35]:

*σ*_0_ = 2.4*Hv* (11)

The mechanical and physical properties of the composites are presented in Table 8. Clearly, due to the existence of nano-sized carbide, all of the Young’s modulus, Vickers hardness, and yield strength values were increased in the C-doped composites. It has been reported that, the hardness and Young’s modulus of Nb_2_C is higher than that of Nb_ss_ [36]. With the addition of C, Nb_ss_ was transformed to Nb_2_C. Based on the rule of mixtures [32], the hardness and Young’s modulus of the Nb_ss_ layer would increase, leading to an increase in yield strength, according to Equation (11).

## 4. Conclusions

Laminated Nb/Nb_5_Si_3_ composites supplemented with C were prepared via spark plasma sintering. Nb_ss_ and γ-Nb_5_Si_3_ were found In the Nb-Si-C composites, and micron-sized Nb_2_C particles and nano-sized Nb_2_C were observed in the Nb_ss_ layer. The formation of Nb_2_C particles might be attributable to the rapid diffusion of C into the Nb foil during sintering, and the formation of nano-sized Nb_2_C could be attributable to C’s solid solubility change in Nb_ss_. Additionally, with the addition of C, the compressive strength of composites at 1400 °C and the fracture toughness increased from 310 MPa and 11.9 MPa·m^1/2^ to 330 MPa and 14.2 MPa·m^1/2^, respectively; the addition of C mainly resulted in solid solution strengthening.

## Figures and Tables

**Table 1:** PDF card number of each XRD phase.

Phase	PDF Card Number
α-Nb_5_Si_3_	00-009-0272
Nb_2_C	00-015-0127
Nb_ss_	00-001-1183
γ-Nb_5_C_3_	03-065-2781

**Table 2:** Thermodynamic data of material in reaction (1).

Materials	$∆H_{298}^{\Theta }/J$	$∆S_{298}^{\Theta }/J\cdot K^{-1}$
Nb	0	36.401
SiC	−73,220	16.610
Nb_5_Si_3_	−451,872	251.040
Nb_2_C	−194,974	64.015

**Table 3:** WDS composition analysis of the micro-areas in Figure 3.

Position	Composition (at%)				Possible Phase
	Nb	Si	C	O	
1	97.8	0.9	0.0	1.3	Nbss
2	62.8	37.2	0.0	0.0	Nb_5_Si_3_
3	62.4	32.1	5.5	0.0	C-poor Nb_5_Si_3_
4	62.7	24.6	12.7	0.0	C-rich Nb_5_Si_3_
5	98.8	0.9	0.0	0.3	Nbss
6	71.1	0.0	28.9	0.0	Nb_2_C
7	69.9	0.0	30.1	0.0	Nb_2_C

**Table 4:** Thermodynamic data of the materials in reaction (4).

Materials	$∆H_{298}^{\Theta }/J$	$S_{298}^{\Theta }/J\cdot K^{-1}$
NbO	−408,777	50.208
C	0	5.732
Nb	0	36.401
CO	−110,541	197.527

**Table 5:** Thermodynamic data of the materials in reactions (6) and (7).

Materials	$∆H_{298}^{\Theta }/J$	$S_{298}^{\Theta }/J\cdot K^{-1}$
Nb_2_C	−194,974	64.015
Si	0	18.828
Nb_5_Si_3_	−451,872	251.040
NbSi_2_	−138,072	69.873
C	0	5.732

**Table 6:** Lattice parameters of Nb_ss_ and α-Nb_5_Si_3_ in the composites.

Composites	a (Nb_ss_)	a (α-Nb_5_Si_3_)	c (α-Nb_5_Si_3_)
Nb-50Si	3.31519	6.56971	11.89522
Nb-40Si-10C	3.31026	6.53410	11.86043

**Table 7:** Volume fractions of each phase and mechanical properties of alloys.

Composites	Actual Compositions (at.%)	Volume Fraction of Nb_ss_/%	Volume Fraction of Nb_5_Si_3_/%	Volume Fraction of Nb_2_C/%	Compressive Strength at 1400 °C/MPa	Fracture Toughness/MPa·m^1/2^
Nb-50Si	Nb-22.1Si-0.5O	41.3	58.7	0	310	11.9
Nb-40Si-10C	Nb-19.2Si-4.9C-0.1O	39.7	56.7	3.6	330	14.2

**Table 8:** Mechanical and physical properties of alloys.

	Nb-50Si	Nb-40Si-10C
Volume fraction of Nb_ss_, *V_f_*	41.3 ± 0.1	39.7 ± 0.1
Radius of Nb_ss_, *a*_0_ (μm)	6.3 ± 0.1	6.1 ± 0.1
Young’s modulus, *E* (GPa)	105.0 ± 2.1	116.0 ± 2.2
Vickers hardness, *Hv* (MPa)	279.0 ± 3.1	307.0 ± 3.1
Yield strength of Nb_ss_, *σ*_0_ (MPa)	670.0 ± 4.1	737.0 ± 4.5
Calculated, Δ*K* (MPa·m^1/2^)	16.8 ± 0.9	17.9 ± 0.8
Fracture toughness, *K_Q_* (MPa·m^1/2^)	11.9 ± 2.9	14.2 ± 3.2

## Data Availability

Not applicable.
